# Unveiling excitons in two-dimensional $\beta$-pnictogens

**DOI:** 10.1038/s41598-024-62094-z

**Published:** 2024-05-22

**Authors:** Marcos R. Guassi, Rafael Besse, Maurício J. Piotrowski, Celso R. C. Rêgo, Diego Guedes-Sobrinho, Andréia Luisa da Rosa, Alexandre Cavalheiro Dias

**Affiliations:** 1 Faculty of Applied Technology and Social Science, Brasília Unified Education Center, Brasília, 70790-075 DF Brazil; 2 Institute of Physics, University of Brasília, Brasília, DF 70919-970 Brazil; 3 Department of Physics, Federal University of Pelotas, PO Box 354, Pelotas, RS 96010-900 Brazil; 4 Karlsruhe Institute of Technology (KIT), Institute of Nanotechnology Hermann-von-Helmholtz-Platz, 76344 Eggenstein-Leopoldshafen, Germany; 5 Chemistry Department, Federal University of Paraná, Curitiba, 81531-980 Brazil; 6 Instituto de Física, Universidade Federal de Goiás, Campus Samambaia, Goiânia, GO 74690-900 Brazil; 7 Institute of Physics and International Center of Physics, University of Brasília, Brasília, DF 70919-970 Brazil

**Keywords:** 2D materials, Pnictogens, Excitons, Density-functional theory, Bethe–Salpeter equation, Chemistry, Energy science and technology, Engineering, Nanoscience and technology, Optics and photonics, Physics

## Abstract

In this paper, we investigate the optical, electronic, vibrational, and excitonic properties of four two-dimensional $\beta$-pnictogen materials—nitrogenene, phosphorene, arsenene, and antimonene—via density functional theory calculations and the Bethe–Salpeter equation. These materials possess indirect gaps with significant exciton binding energies, demonstrating isotropic behavior under circular light polarization and anisotropic behavior under linear polarization by absorbing light within the visible solar spectrum (except for nitrogenene). Furthermore, we observed that Raman frequencies red-shift in heavier pnictogen atoms aligning with experimental observations; simultaneously, quasi-particle effects notably influence the linear optical response intensively. These monolayers’ excitonic effects lead to optical band gaps optimized for solar energy harvesting, positioning them as promising candidates for advanced optoelectronic device applications.

## Introduction

Many research efforts have focused on innovating new materials and unraveling their unique physical properties to facilitate the development of advanced nanodevices that outperform current models in size, speed, functionality, and energy efficiency. The groundbreaking synthesis of graphene, as highlighted in Novoselov’s work^1^, marked the beginning of an exciting era in exploring two-dimensional materials. These materials are essential to miniaturizing devices while improving their thermal dissipation capabilities^2^. However, graphene, despite its potential, faces specific challenges. Its low inherent spin-orbit interaction and semimetallic nature with a zero band gap^3^ pose significant limitations. Even the strain performing a band gap in graphene requires extremely high strain levels, which is only sometimes feasible^4^, has spurred interest in other two-dimensional (2D) materials. Additionally, even though group IV materials are extensively studied, they also suffer from the limitation of having a zero band gap^5^, which is a drawback for various applications.

Materials from Group V, known as pnictogens, are emerging as viable candidates for applications as thermoelectrics, semiconductors, and in the nonlinear optic field^6^. Particularly noteworthy is the synthesis of structures such as honeycomb-$\beta$ arsenene^7–9^, phosphorene^10^, and antimonene^9,11,12^, along with the more recent development of nitrogenene under high-temperature and pressure conditions^13,14^. Chemical elements in this Group exhibit significantly greater intrinsic spin-orbit coupling when compared to graphene and Group IV materials. Furthermore, their band gaps are not only adjustable but also display indirect band gaps, with ranges typically lying between 1 and 3 eV, as predicted within the DFT-PBE approximation^15^. Although monolayer antimonene is characterized as a semiconductor featuring a sizeable indirect band gap of 2.28 eV, theoretical studies suggest that this band gap may progressively diminish to zero as the number of layers increases^16^.

Theoretical calculations of band gaps using the GW approach have revealed that the electronic band gaps of these materials span a spectrum ranging from near-infrared to visible light. Specifically, for arsenene, the band gap values are 2.56 eV for the buckled form and 1.51 eV for the puckered variant^17^. The Bethe–Salpeter approach has also demonstrated notable exciton binding energies, measuring at ${690}\,\hbox {meV}$ for buckled arsenene and ${484}\,\hbox {meV}$ for puckered arsenene^17^. Despite a plethora of experimental^7,9,11^ and theoretical^15,18–22^ studies on 2D pnictogens. However, neither of these investigations delves into the mechanisms by which each pnictogen atomic species can alter the exciton binding energy and the excitonic ground state’s nature, whether directly or indirectly.

This work shows the potential of 2D pnictogens (nitrogenene, phosphorene, arsenene, and antimonene) through their thermodynamic stability at low temperatures, given their vibrational behavior as Raman active modes, which are compared with existing experimental results. Except for nitrogenene (with a large electronic band gap), all the systems are characterized as semiconductors, so their electronic band gaps align with the solar absorption spectrum. Notably, we found that quasi-particle phenomena are pronounced due to their significant exciton binding energies, and there is isotropic behavior under circular light polarization. In contrast, anisotropy is exhibited when subjected to linear polarization. When excitonic effects are factored in, all structures demonstrate semiconductor-like optical band gaps within the solar emission range. Therefore, the incorporation of excitonic effects illuminates the potential of 2D $\beta$-pnictogen monolayers in advancing solar harvesting technologies and nanoelectronic devices for future industrial innovations, which are more closely with the optimal range for maximal solar energy harvesting efficiency, specifically for the aforementioned semiconductor-like pnictogen group.

## Computational methods

### Ab initio calculations for geometry optimzation

**Figure 1:**
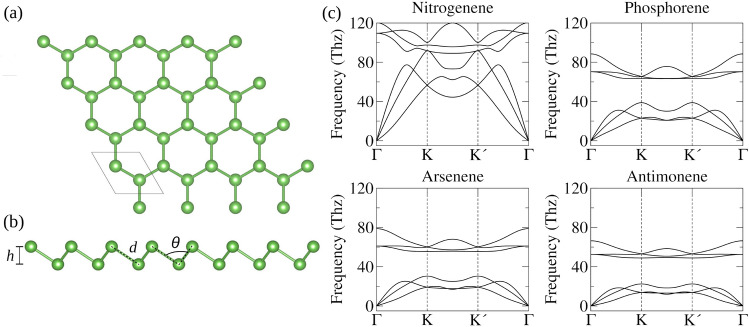
Relaxed crystal structure of $\beta$-pnictogen monolayers: (**a**) top view and (**b**) side view. The side view includes key structural parameters employed to describe the monolayers. In (**c**), phonon dispersion curves of $\beta$ phase of nitrogenene, phosphorene, arsenene, and antimonene monolayers calculated using PBE.

Our 2D honeycomb-$\beta$ pnictogens monolayers based on nitrogenene, phosphorene, arsenene, and antimonene are depicted in the hexagonal structure shown in Fig. 1a, b, which were determined as geometries for all $\beta$-monoelemental single-layered nanostructures^15^. The simulations were based on density functional theory (DFT) within the framework of the generalized gradient approximation (GGA), explicitly employing the Perdew–Burke–Ernzerhof (PBE) exchange-correlation functional^23^. The Kohn–Sham (KS) equations pivotal to our analysis were solved using the projector augmented wave (PAW) method^24,25^, as implemented in the Vienna ab initio Simulation Package (VASP)^26,27^. Structure optimizations in our simulations were meticulously conducted, ensuring the minimization of stress tensor and atomic forces until the atomic forces fell below 0.01 eV Å$^{-1}$.

For our calculations, we employed a plane-wave cutoff energy set at $2\times$ ENCUT$_i$, with $i={\hbox {N}, \hbox {P}, \hbox {As}, \hbox {Sb}}$ and ENCUT$_i$ representing the highest recommended cutoff energy for each pnictogen, being ENCUT$_{N}={420.902}\,\hbox {eV}$, ENCUT$_{P}={255.040}\,\hbox {eV}$, ENCUT$_{As}={208.702}\,\hbox {eV}$ and ENCUT$_{Sb}={172.069}\,\hbox {eV}$. A reduced cutoff of $1.125\times$ENCUT$_i$ was used for computing other properties. Details of the PAW projector used for each pnictogen can be found in the support information (SI), Table S1. In executing the KS self-consistent cycle, we adhered to a stringent total energy convergence criterion of $10^{-6}\,\hbox {eV}$. To eliminate any spurious interactions between the monolayer and its periodic images in the *z*-direction, we incorporated a vacuum layer with a thickness of 16 Å in each monolayer unit cell. For all calculations, **k**-meshes were automatically generated utilizing the Monkhorst-Pack method^28^, ensuring a **k**-points density of 40 Å$^{-1}$ in the directions of the in-plane lattice vectors.

### Phonons calculations

Phonon calculations involved $3\times 3\times 1$ monolayer supercells with the same cutoff energies and **k**-points densities used in the electronic properties calculations. The off-resonance Raman activity was determined using the method developed by Porezag and co-workers^29^, considering the phonon vibration modes at $\Gamma$. The computational implementation proposed by Fonari and Stauffer^30^ was employed for these calculations.

### Tight-binding parameters

For solving the Bethe–Salpeter equation (BSE), we employed the Rytova-Keldysh 2D Coulomb Potential (V2DRK)^31^, considering the monolayers in a vacuum setting. Each monolayer’s effective dielectric screening parameter was determined by averaging the in-plane diagonal components of the macroscopic dielectric constant, with a unit cell vacuum spacing of 24 Å in the non-periodic direction. Our calculations were performed with a 120 Å$^{-1}$ density of **k**-points and a smearing factor of ${0.05}\,\hbox {eV}$ to accurately derive the real and imaginary parts of the dielectric function. The optical properties were calculated at the Independent Particle Approximation (IPA) and BSE levels, considering the lowest 6 conduction bands and the highest 6 valence bands. Additional information about the BSE parameters is available in Table S2.

To overcome the band gap underestimation by semi-local functionals^32,33^, we augmented our approach with the hybrid range-separated Heyd–Scuseria–Ernzerhof (HSE06) exchange-correlation functional^34,35^. The spin-orbit coupling (SOC) effects were comprehensively integrated into PBE and HSE06 functionals through the second variational method^36,37^. Based on that, the linear optical response of the materials was meticulously computed to include excitonic effects, achieved through solving the Bethe–Salpeter equation (BSE)^38,39^ and also without these effects (independent particle approximation – IPA) by employing the WanTiBEXOS package^39^; more details in Supplementary Materials. We first created a maximally localized Wannier function tight-binding (MLWF-TB) Hamiltonian to conduct these evaluations derived from HSE06+SOC DFT simulations through the Wannier90 package^40^, emphasizing each pnictogen’s *s* and *p* projections.

## Results

The dynamic stability of the systems was investigated via phonon dispersion by combining VASP and the Phonopy package^41^ based on the density functional perturbation theory (DFPT) method. As depicted in Fig. 1c, our systems present an absence of imaginary frequencies, which indicates their dynamic stability at low temperatures. For phosphorus (P), arsenic (As), and antimony (Sb), the three acoustic phonon branches are observed below ${40}\,\hbox {THz}$. In contrast, for nitrogen (N), these branches extend up to a range between ${60}\,\hbox {THz}$ and ${90}\,\hbox {THz}$. One observes that the higher frequency range and the absence of a gap between the acoustic and optical modes in the N system suggest distinct vibrational characteristics compared to the other pnictogens. This variation could affect these materials’ thermal and electronic properties, potentially influencing their suitability for various applications.

**Figure 2:**
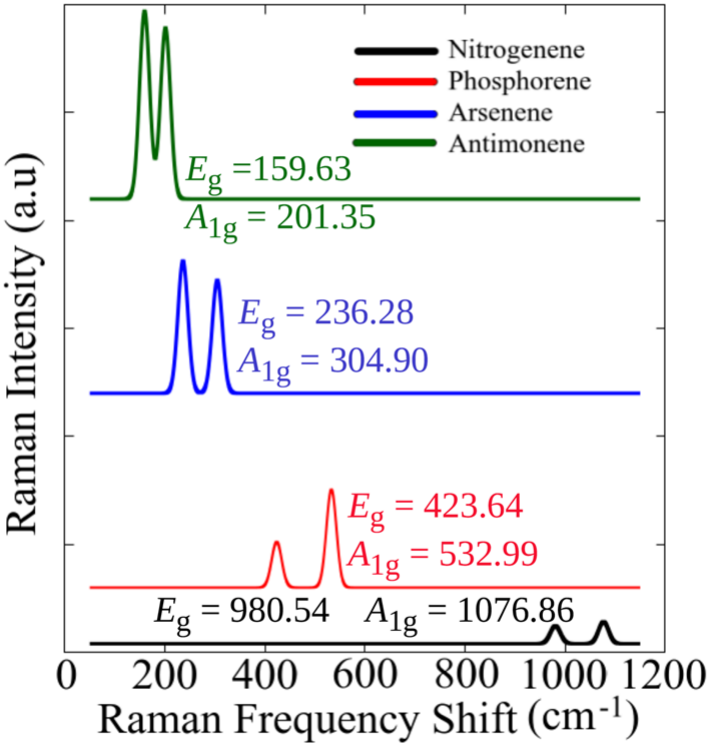
The Raman spectrum of nitrogenene is represented by black curves, phosphorene by red curves, arsenene by blue curves, and antimonene by green curves. The values of $E_{g}$ and $A_{1g}$ modes are numerically highlighted for each system.

**Table 1:** Relaxed structural parameters of nitrogenene, phosphorene, arsenene, and antimonene monolayers: the lattice parameter ($a_0$), buckling height (*h*), bond length (*d*), and bond angle ($\theta$). Our results are compared with previous literature reports in bold.

System	$a_0$	*h*	*d*	$\theta$
	Å	Å	Å	$^\circ$
Nitrogenene	2.29	0.70	1.50	100.07
**Nitrogenene** ^15^	**2.30**	**0.70**	**1.50**	
Phosphorene	3.28	1.24	2.27	92.82
**Phosphorene** ^22^	**3.28**	**1.24**	**2.26**	
**Phosphorene** ^15^	**3.28**	**1.24**	**2.26**	
Arsenenene	3.61	1.40	2.51	91.97
**Arsenenene** ^18^	**3.63**	**1.40**	**2.52**	**91.95**
**Arsenenene** ^15^	**3.61**	**1.40**	**2.51**	
**Arsenenene** ^7^	**3.61**	**1.45**		
Antimonene	4.11	1.64	2.89	90.81
**Antimonene** ^18^	**3.98**	**1.61**	**2.80**	**90.29**
**Antimonene** ^15^	**4.12**	**1.65**	**2.89**	

In Fig. 2, we present the Raman spectra of different systems, highlighting their $E_{g}$ and $A_{1g}$ modes. The $E_{g}$ mode, which is double-degenerate, consistently exhibits a lower frequency than the $A_{1g}$ mode, regardless of the pnictogen’s chemical composition. Notably, for nitrogenene and phosphorene, the $A_{1g}$ mode shows higher Raman activity. In contrast, for arsenene and antimonene, the double-degenerate $E_{g}$ mode takes precedence regarding Raman intensity. Thus, it is evident that the Raman frequencies depend on the chemical composition of a substance. As the substance contains heavier atoms, the Raman-active modes red-shift, having lower frequencies. Our research on the Raman spectrum confirms the observations made by Deng et al.^19^ in their study on 2D honeycomb-$\beta$ phosphorene.

Experimental values of Raman spectroscopy for arsenene have been reported to be ${197.2}\,\hbox {cm}^{-1}$ and ${256.5}\,\hbox {cm}^{-1}$, corresponding to the in-plane E$_{g}$ and out-of-plane A$_{1g}$ modes, respectively^8^. However, our study has found modes at ${236.28}\,\hbox {cm}^{-1}$ and ${304.90}\,\hbox {cm}^{-1}$. The difference between our findings and the reported values may be because we considered free-standing layers, which shift the frequencies to higher values. Additionally, in the experiment, the thickness of the nanosheets varied in the range of ${2.0}-{4.0}\,\hbox {nm}$, corresponding to 6-12 layers. However, we have only calculated the Raman shifts for a monolayer. Conversely, experiments involving antimonene have indicated a band at ${113.6}\,\hbox {cm}^{-1}$ associated with an in-plane vibrational mode and another originated from an out-of-plane vibrational mode observed at ${150.6}\,\hbox {cm}^{-1}$^16^. Our findings of ${159.63}\,\hbox {cm}^{-1}$ and ${201.35}\,\hbox {cm}^{-1}$ has a similarly difference from Arsenene case. This suggests that our results for Arsenene and Antimonene relatively agree well with the reported experimental results^8,16^.

The lattice constant ($a_0$), buckling height (*h*), and bond length (*d*) values exhibit an increasing trend with the atomic number of the elements; see Table 1. The concurrent increase in $a_0$, *h*, and *d* suggests a lack of preferential orientation, whether in-plane or out-of-plane, as the atomic size expands. This observation is further reinforced by the minimal variation in the bond angle ($\theta$). However, it is observed that the angle $\theta$ decreases with an increasing atomic number. Furthermore, the $\beta$ monolayer phase is not unique to Group-V materials, as it has also been observed in Group-IV elements^42^. While the bond lengths between these two groups of materials are comparable, a significant difference is evident in the degree of vertical distortion, as indicated by the buckling height (*h*). For instance, in silicene, germanene, and stanene-materials belonging to Group IV-the reported buckling heights range from 0.44 Å  to  0.85 Å^43^. This range is notably different from that of the Group-V materials studied here, which exhibit buckling heights ranging from 0.69 Å  to  1.64 Å. We have shown that despite groups IV and V share the same crystal phase, the buckling height is larger at Group V elements.

**Figure 3:**
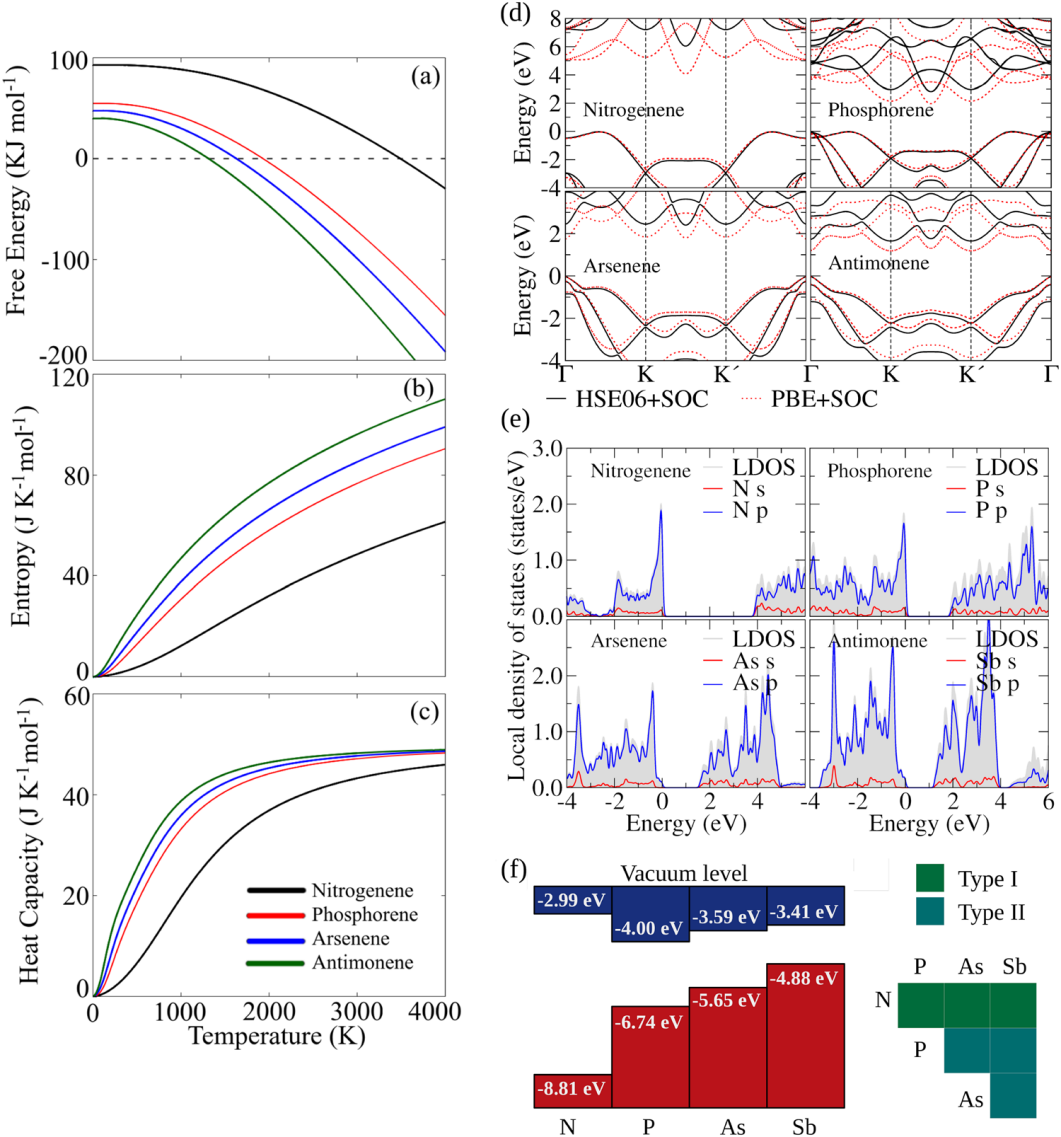
Thermodynamic properties of 2D pnictogens: (**a**) Gibbs Free Energy, (**b**) entropy, and (**c**) heat capacity at constant volume. (**d**) Comparison of DFT-PBE+SOC and DFT-HSE06+SOC electronic band structures for nitrogenene, phosphorene, arsenene, and antimonene monolayers with valence band maxima set to zero energy. (**e**) The DFT-PBE electronic local density of states (LDOS) for nitrogenene, phosphorene, arsenene, and antimonene monolayers with projections onto angular momentum states. Valence band maxima are set to zero energy. (**f**) The natural band edge energies of nitrogenene (N), phosphorene (P), arsenene (As), and antimonene (Sb) obtained with DFT-HSE06+SOC with the classification of heterojunction types based on Anderson’s rule.

Based on phonon dispersion data, we calculated key thermal properties of monolayer pnictogens within the harmonic approximation framework. These properties include the Gibbs free energy, entropy, and heat capacity at constant volume, as detailed in Fig. 3a, b, c. We observed a trend where free energies decrease with an increase in atomic number, aligning with expectations considering the weaker binding energies. Nitrogenene demonstrates a significantly larger deviation in free energy than other pnictogens. The negative values of free energy are attained at approximately ${1900}\,\hbox {K}$ for phosphorene, ${1600}\,\hbox {K}$ for arsenene, and ${1300}\,\hbox {K}$ for antimonene. For nitrogenene, this threshold is reached around ${3500}\,\hbox {K}$, consistent with the experimental data on its high-temperature synthesis^13,14^.

Entropy exhibits an increasing trend from nitrogen (N) to antimony (Sb), with nitrogenene showing a comparatively gradual increase with temperature. The chemical trends of heat capacities mirror those of entropy, with a rapid rise observed up to ${1000}\,\hbox {K}$ for phosphorene, arsenene, and antimonene, eventually plateauing around ${3000}\,\hbox {K}$ at the Dulong-Petit limit. In contrast, nitrogenene’s heat capacity shows a slower temperature-dependent increase. For instance, at ${1000}\,\hbox {K}$, its heat capacity is less than ${60}\%$ of that of the other structures, and it remains about ${5}\,\hbox {J}\,\hbox {K}^{-1}\,\hbox {mol}^{-1}$ below the asymptotic limit even at ${4000}\,\hbox {K}$.

Regarding the structural characteristics in the equilibrium of these monolayers, we analyzed equilibrium parameters, including the lattice constant ($a_0$), buckling height (*h*), bond length (*d*), and bond angle ($\theta$), all of which are detailed in Fig. 1b. The stability of these pnictogens was also evaluated by calculating the cohesive energy ($E_\text {coh}$), defined as $E_\text {coh} = (E_\text {tot}^{\text {mono}} - nE_\text {tot}^{\text {free-atom}})/n$, where $E_\text {tot}^{\text {mono}}$ represents the total energy of the monolayer, $E_\text {tot}^{\text {free-atom}}$ is the total energy of the free atom, and *n* denotes the total number of atoms in the structure. For instance, we found that nitrogene, phosphorene, arsenene, and antimonene present $E_\text {coh} = -3.582, -3.458, -2.971$, and ${-2.629}\,\hbox {eV}/\hbox {atom}$, respectively. The $E_\text {coh}$ results reveal a trend indicating decreasing bonding strength from nitrogen (N) to antimony (Sb), correlating with larger bond lengths in the respective systems.

### Electronic properties

To analyze the electronic properties of 2D pnictogens, these materials’ conduction and valence bands were plotted in Fig. 3d. Noticeable differences arise between the two employed functionals, HSE06+SOC and PBE+SOC, primarily due to the smaller band gaps obtained with PBE+SOC – a characteristic feature of this semilocal functional. Nevertheless, the general features of the band structures remain consistent for both functionals, featuring an indirect band gap in both cases. The fundamental band gap for nitrogenene is ${5.78}\,\hbox {eV}$, as seen in Table 2, with a direct gap of ${5.96}\,\hbox {eV}$. This result suggests that the material is insulating. Both values for the energy gap will decrease significantly when excitons are considered. However, as indicated by the data in Table 2, one immediately realizes a discernible trend of decreasing band gaps with the increasing atomic number of the materials. Therefore, in contrast to nitrogenene, the band gaps of these materials suggest potential applications in semiconductor devices, as both indirect and direct gaps are lower than ${3}\,\hbox {eV}$. Evidence of the formation of monolayer arsenene on Ag(111) has been reported in Ref.^7^. Our results for free-standing arsenene are in fair agreement with the experimental band gap reported in Ref.^8^.

Aiming to analyze a potential application of these monolayers in heterojunctions, the local density of states (LDOS) for the monolayers – as shown in Fig. 3e—were calculated, which revealed a predominance of *p* states near the band edges and a smaller contribution of *s* states. Thus, we advance in applying Anderson’s rule to estimate the heterojunction band offsets to understand the band alignment of heterojunctions owing to the weak interactions in vertically stacked layers^44^. Anderson’s rule states that when constructing an energy band diagram, the vacuum levels of the two semiconductors on either side of the heterojunction should be aligned at the same energy. Here, the energy positions of the valence band maximum (VBM) and conduction band minimum (CBM) were obtained from the vacuum level as defined by the electrostatic potential value in the center of the vacuum region (see details in Ref^45^). Figure 3f reveals that the relative position of VBM is higher when going from N to Sb. This is correlated with the increase of the atomic *s* and *p* orbital energies. A similar trend is observed in the CBM, except for N, which has a higher CBM than all the remaining systems. Finally, we suggest that junctions with a type-II staggered gap are formed, except for heterostructures with N, where junctions with a straddling gap (type I) are seen, an interesting feature for solar harvesting devices.

### Excitonic and optical properties

**Figure 4:**
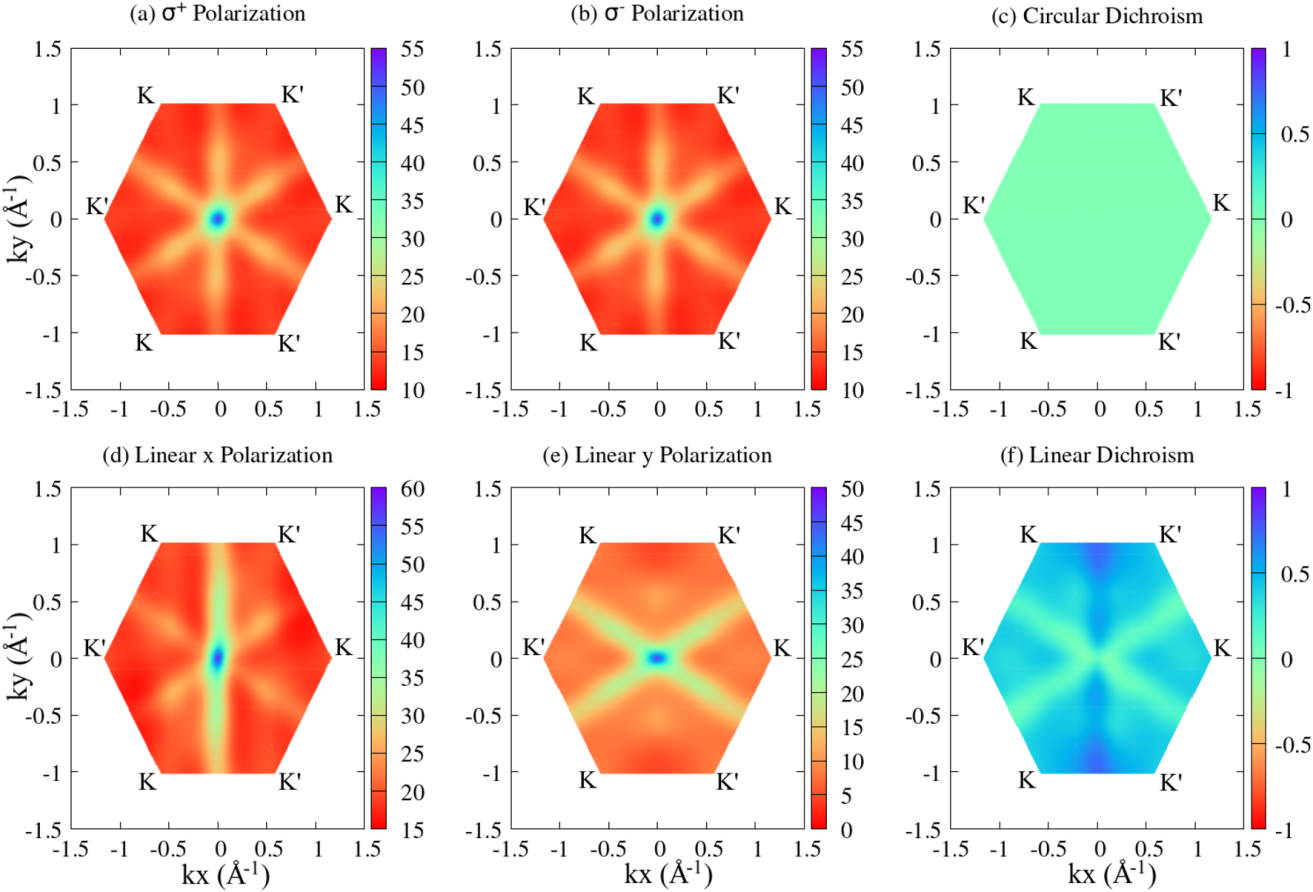
Arsenene’s optical activity in the first Brillouin Zone with (**a**) and (**b**) circular light polarization and (**d**) and (**e**) linear light polarization. Additionally, circular optical dichroism is depicted in (**c**), while linear optical dichroism is shown in (**f**). All calculations were carried out using DFT-HSE06+SOC and MLWF-TB parametrization.

For a deeper understanding of the excitonic properties of these monolayers, Table 2 provides a comparison between the direct band gap $E^{d}_{g}$ and the direct exciton ground state $Ex^{d}_{gs}$, representing the lowest exciton energy for states where the electron and hole pairs share the same momentum **k**. The exciton binding energy $Ex_{b}$ is defined as the difference between $E^{d}_{g}$ and $Ex^{d}_{gs}$. The trend seen for the $Ex_{b}$ is in agreement with the direct band gap and decreases as the atomic mass of the pnictogen increases from N to Sb. Remarkably, the exciton binding energies are notably larger than those found in other 2D materials, such as TMDs, where typical values range from ${0.1} \hbox { to}\,{0.5}\,\hbox {eV}\,$^37^.

**Table 2:** Excitonic properties obtained by MLWF-TB+BSE: fundamental band gap, $E_{g}$, direct band gap, $E^{d}_{g}$, direct exciton ground state, $Ex^{d}_{gs}$, and exciton binding energy, $Ex_{b}$, calculated as $E^{d}_{g}-Ex^{d}_{gs}$.

System	$E_{g}$ (eV)	$E^{d}_{g}$ (eV)	$Ex^{d}_{gs}$ (eV)	$Ex_{b}$ (eV)
Nitrogenene	5.78	5.96	2.38	3.59
Phosphorene	2.67	2.91	1.33	1.57
Arsenenene	2.06	2.50	1.43	1.06
Antimonene	1.43	1.78	1.21	0.57

We found that nitrogenene exhibits an $Ex_{b}$ of ${3.59}\,\hbox {eV}$, indicating substantial quasi-particle effects. Similarly, phosphorene (${1.57}\,\hbox {eV}$) and arsenene (${1.06}\,\hbox {eV}$) demonstrate considerable binding energies, while antimonene exhibits an $Ex_{b} = {0.57}\,\hbox {eV}$, which is similar to typical 2D materials. Notably, $Ex_{b}$ is highly sensitive to the surrounding dielectric environment^37,46,47^ suggesting the potential for optical band gap tuning by altering the chemical environment. This sensitivity opens the path for maximizing the applicability of the investigated 2D pnictogens in specific target applications.

To provide a solid understanding of the interaction of those monolayers with light, the responses to the circular and linear light polarizations, as well as the circular and linear optical dichroism, are depicted in Fig. 4, which presents the single-particle optical activity within the first BZ for arsenene as a representative example. Comparable data for the remaining systems is available in the SI. In panels (a) and (b), the monolayer exhibits isotropy in response to circular light polarization. This is further emphasized in panel (c), where circular dichroism remains at 0 throughout the BZ. Conversely, the As-monolayer displays anisotropy under linear light polarization, revealing distinct responses for $\hat{x}$ (panel (d)) and $\hat{y}$ (panel (e)) polarizations and resulting in non-zero linear dichroism, as depicted in panel (f). Regardless of the direction of light polarization, it is evident that optical absorption is more pronounced at the BZ center around $\Gamma$ and tends to 0 around the *K* and $K'$ valleys. Notably, this monolayer exhibits optical activity extending from the $\Gamma$-point towards the region between the *K* and $K'$ valleys. Under circular polarization, activity is observed in all directions inside the hexagonal BZ. In the case of linear polarization, specific directions exhibit high optical activity, depending on $\hat{x}$ and $\hat{y}$ polarizations. Similar optical behaviors are seen for the other 2D pnictogens.

**Figure 5:**
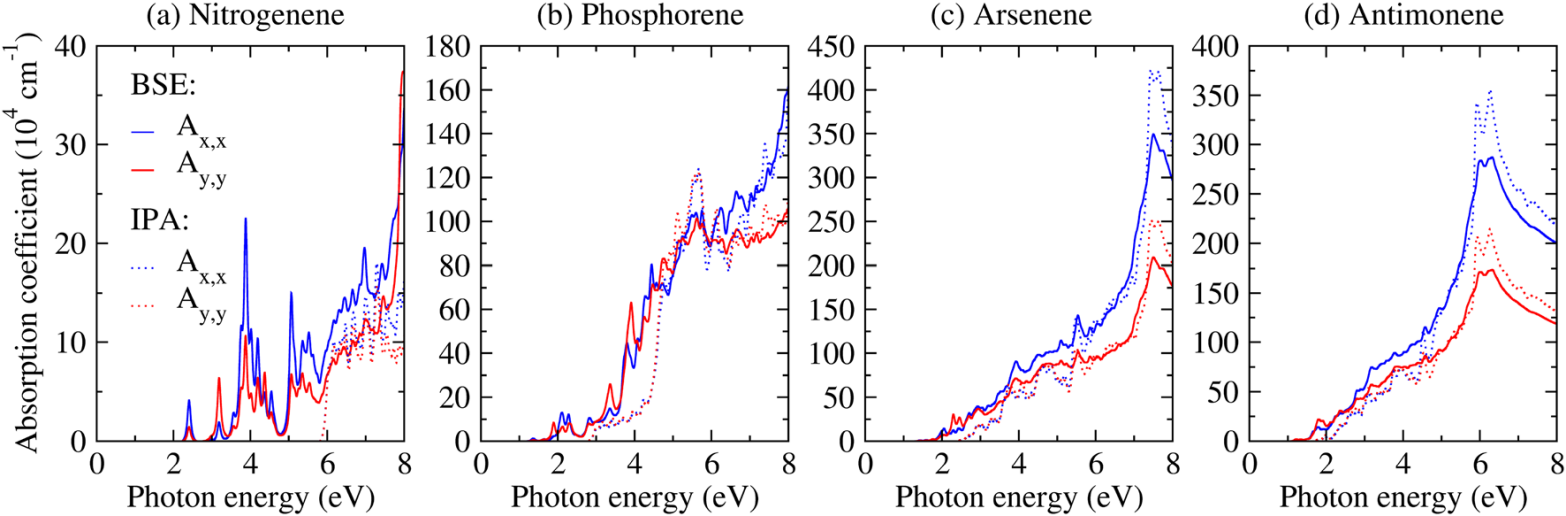
Absorption spectrum obtained through MLWF-TB approach at both the BSE level (solid curves) and the IPA level (dashed curves) for (**a**) nitrogenene, (**b**) phosphorene, (**c**) arsenene, and (**d**) antimonene. The blue and red curves represent linear light polarizations along the $\hat{x}$ and $\hat{y}$ directions, respectively.

The importance of the excitonic effects is also analysed in the absorption spectra results by comparing the IPA (dashed curves) and BSE (solid curves) levels, as depicted in Fig. 5 for all studied monolayers. The linear light polarizations along $\hat{x}$ (blue curves) and $\hat{y}$ (red curves) directions are considered. All direct excitonic ground states ($Ex^{d}_{gs}$) are bright, indicating optical response in the absorption spectrum. Additionally, the optical band gaps at the IPA level coincide with the direct band gaps. A notable distinction emerges when comparing the BSE and IPA optical responses. Firstly, the optical band gap experiences significant changes due to the large values of exciton binding energy. Furthermore, the spectrum shape reveals enhanced optical anisotropy using MLWF-BSE level, particularly for higher-energy optical transitions, shown by the absorption coefficient different values along $\hat{x}$ and $\hat{y}$ linear light polarizations. The origin of this anisotropy can be attributed to the buckling of the monolayers, which leads to enhanced bond deviations along the $\hat{x}$ and $\hat{y}$ directions within the crystal structure. Additionally, it is influenced by the orbital symmetry of the electron and hole states involved in each optical transition. This effect is further amplified when excitonic effects are considered, as each absorption peak arises from a linear combination of several electron-hole pairs across the entire Brillouin zone, as accounted for by the Bethe–Salpeter equation (BSE). This heightened anisotropy is evident in arsenene and antimonene, as illustrated in Fig. 5c, d.

Therefore, we found that all systems exhibit light absorption within the solar spectrum emission region (0.5–4.0 eV) at the BSE level^48^. However, at the IPA level, only nitrogenene refrains from absorption in this region. The comparison between BSE and IPA optical band gaps is crucial, considering the substantial influence of the external dielectric environment on excitonic optical band gaps^37,46,47^ As our calculations involve free-standing monolayers in a vacuum, we establish the lower limit for the optical band gap from our calculations and the upper limit from IPA approximations, where exciton binding energies approach ${0}\,\hbox {eV}$. This establishes a range for tuning the optical band gap in these monolayers. The high refractive indices found in the UV region are suggested in Refs.^11,49^ could allow its application in UV absorbers.

## Conclusions

We have identified several key features relevant to technological and industrial applications in our theoretical characterization of 2D $\beta$-pnictogen monolayers, employing DFT and MLWF-BSE formalism. All investigated structures demonstrate thermodynamic stability at low temperatures, a crucial factor for device reliability, evidenced by the absence of imaginary frequencies in their phonon dispersions. The Raman-active optical vibrational modes align with experimental findings, indicating their potential for industrial-scale spectroscopic characterization. Notably, except for nitrogenene with its large electronic band gap, phosphorene, arsenene, and antimonene exhibit semiconductor properties with electronic band gaps within the solar absorption spectrum. This positions them as promising materials for photovoltaic applications, where efficient solar energy conversion is essential. Nitrogenene’s band offsets suggest a type-I band alignment in heterojunctions, potentially valuable for optoelectronic devices where controlled charge carrier confinement is desired. In contrast, heterojunctions involving other pnictogens exhibit a type-II band alignment, making them highly suitable for solar energy harvesting devices due to their enhanced charge separation efficiency. The pronounced quasi-particle effects in these monolayers, as indicated by robust exciton binding energies, are particularly significant. The materials’ isotropic response under circular light polarization and anisotropic response under linear polarization point to applications in polarization-sensitive photodetectors and optical modulators. After considering excitonic effects, all structures display semiconductor-like optical band gaps within the solar emission region. Remarkably, phosphorene, arsenene, and antimonene have optical band gaps that align closely with the peak of solar harvesting efficiency, making them highly attractive for next-generation solar cells.

## Supplementary Information

Supplementary Information.

## Data Availability

The raw data used and/or analyzed during the current study are available on our Git repository https://github.com/ac-dias/2d-pnictogens.
