# Supplementary material for: Unveiling excitons in two-dimensional β-pnictogens
Source: Sci Rep. 2024 May 22;14:11710. doi: 10.1038/s41598-024-62094-z (PMC11636924; doi:10.1038/s41598-024-62094-z)
Supplement: Supplementary file 1 — Supplementary Information. [file 41598_2024_62094_MOESM1_ESM.pdf]

Supporting Information: Unveiling Excitons in Two-Dimensional β-Pnictogens

Marcos R. Guassi,[†] Rafael Besse,[‡] Maurício J. Piotrowski,[¶] Celso R. C. Rêgo,[*,§] Diego Guedes-Sobrinho,[‖] Andréia L. da Rosa,[⊥] and Alexandre C. Dias[#]

†*Faculty of Applied Technology and Social Science, Brasília Unified Education Center, Brasília* 70790-075, *DF, Brazil*

‡*Institute of Physics, University of Brasília, Brasília* 70919-970, *DF, Brazil*

¶*Department of Physics, Federal University of Pelotas, PO Box* 354, *Pelotas, RS,* 96010-900, *Brazil*

§*Karlsruhe Institute of Technology (KIT), Institute of Nanotechnology, Eggenstein-Leopoldshafen, Germany*

‖*Chemistry Department, Federal University of Paraná, Curitiba, PR, 81531-980, Brazil*

⊥*Instituto de Física, Universidade Federal de Goiás, Campus Samambaia, 74690-900, Goiânia, GO, Brazil.*

#*Institute of Physics and International Center of Physics, University of Brasília, Brasília* 70919-970, *DF, Brazil*

E-mail: celso.rego@kit.edu

Contents

1 Computational Technical Details

Table S1: Essential information for the chosen PAW projectors (POTCAR), which encompasses key details such as the PAW-PBE projector name, the creation date, the count of valence electrons (Z_{val}), the electron configuration of valence states, and the maximum recommended cutoff energy (ENMAX), for each of the selected chemical species.

Element	POTCAR PAW-PBE	Date POTCAR	Z_{val}	Valence	ENMAX (eV)
N	N_GW	04/10/2007	5	$2s^2\ 2p^3$	420.902
P	P_GW	03/19/2012	5	$3s^2\ 3p^3$	255.040
As	As_GW	03/20/2012	5	$4s^2\ 4p^3$	208.702
Sb	Sb_GW	03/21/2012	5	$5s^2\ 5p^3$	172.069

2 POSCAR from Optimized Structures

```
Nitrogenene honeycomb buckled monolayer
1.00000000000000
 2.2944985944474388   0.0000000000000000   0.0000000000000000
-1.1472492972237192   1.9870940717391694   0.0000000000000000
 0.0000000000000000   0.0000000000000000  16.0000000000000000
N
2
Direct
 0.6666666666666643   0.3333333333333357   0.4782218539462590
 0.3333333333333357   0.6666666666666643   0.5217781460537410
```

```
Phosphorene honeycomb buckled monolayer
1.00000000000000
 3.2823434942051457   0.0000000000000000   0.0000000000000000
-1.6411717471025726   2.8425928499282360   0.0000000000000000
 0.0000000000000000   0.0000000000000000  16.0000000000000000
P
2
Direct
 0.6666666666666643   0.3333333333333357   0.4611828390103057
 0.3333333333333357   0.6666666666666643   0.5388171609896943
```

Arsenene honeycomb buckled monolayer
1.00000000000000

3.6068925389629594	0.0000000000000000	0.0000000000000000
-1.8034462694814795	3.1236605674624749	0.0000000000000000
0.0000000000000000	0.0000000000000000	16.0000000000000000

As
2
Direct

0.6666666666666643	0.3333333333333357	0.4563431356091456
0.3333333333333357	0.6666666666666643	0.5436568643908544

Antimonene honeycomb buckled monolayer
1.00000000000000

4.1133665496711673	0.0000000000000000	0.0000000000000000
-2.0566832748355837	3.5622799270923751	0.0000000000000000
0.0000000000000000	0.0000000000000000	16.0000000000000000

Sb
2
Direct

0.6666666666666643	0.3333333333333357	0.4486253983738564
0.3333333333333357	0.6666666666666643	0.5513746016261436

3 PBE *vs* PBE+SOC Band Structures

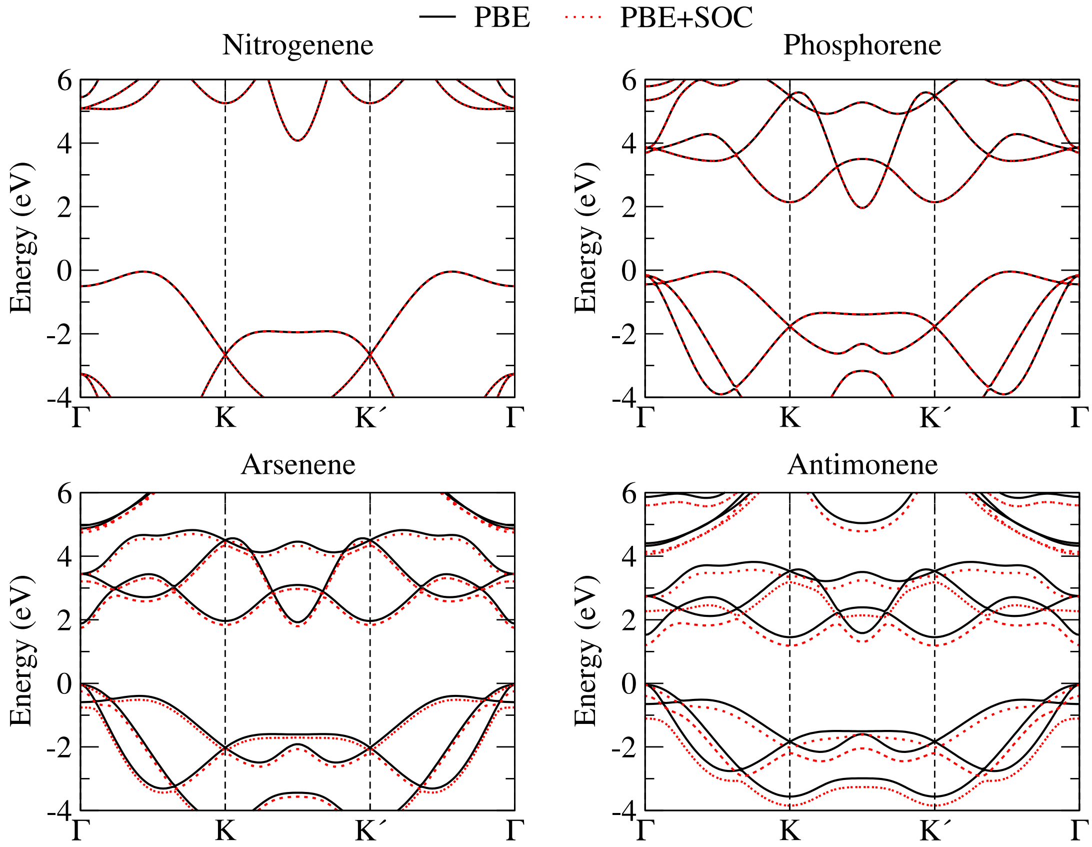

Figure S1: The electronic band structures of nitrogenene, phosphorene, arsenene, and antimonene monolayers are compared using both DFT-PBE and DFT-PBE+SOC methods. The valence band maxima are normalized to zero energy for comparison.

4 Single Particle Optics in BZ

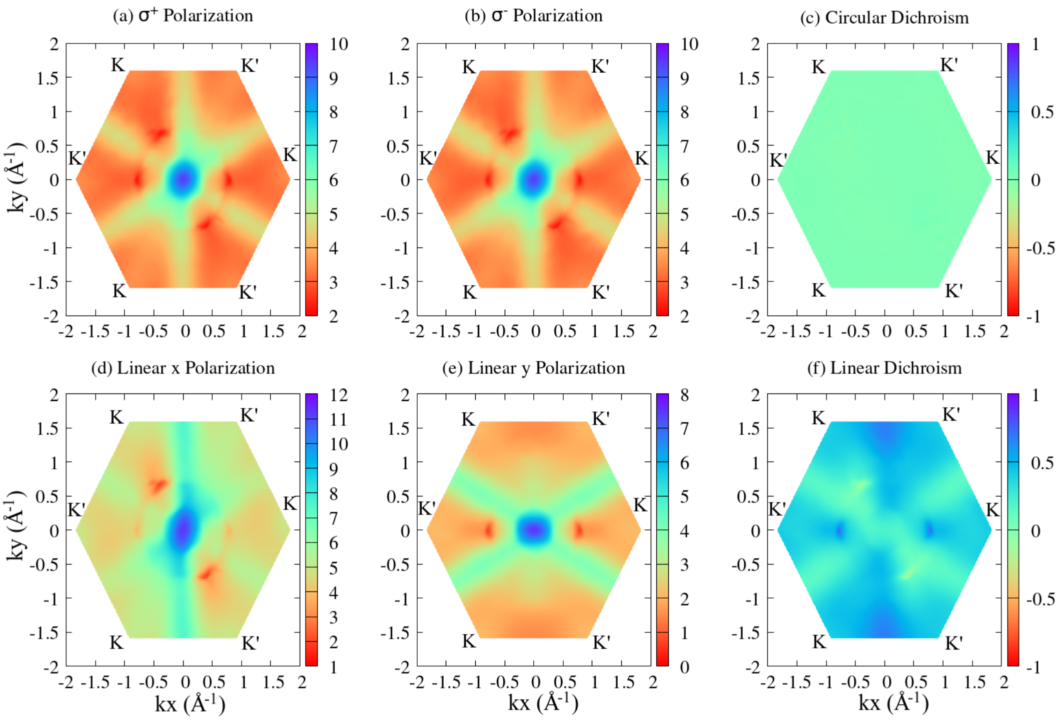

Figure S2: Optical activity of nitrogenene in the first Brillouin Zone (BZ) with circular light polarization in (a) and (b), linear light polarization in (d) and (e), and circular (c) and linear (f) optical dichroism. All calculations were conducted using DFT-HSE06+SOC and MLWF-TB parametrization.

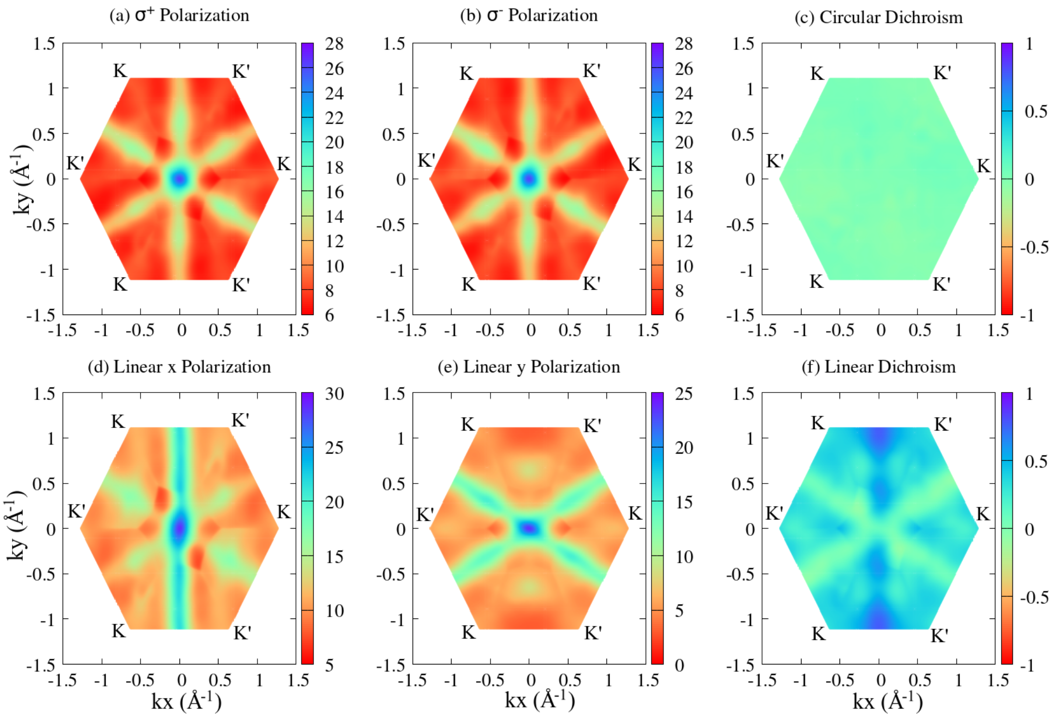

Figure S3: Optical activity of phosphorene in the first BZ with circular light polarization in (a) and (b), linear light polarization in (d) and (e), and circular (c) and linear (f) optical dichroism. All calculations were conducted using DFT-HSE06+SOC and MLWF-TB parametrization.

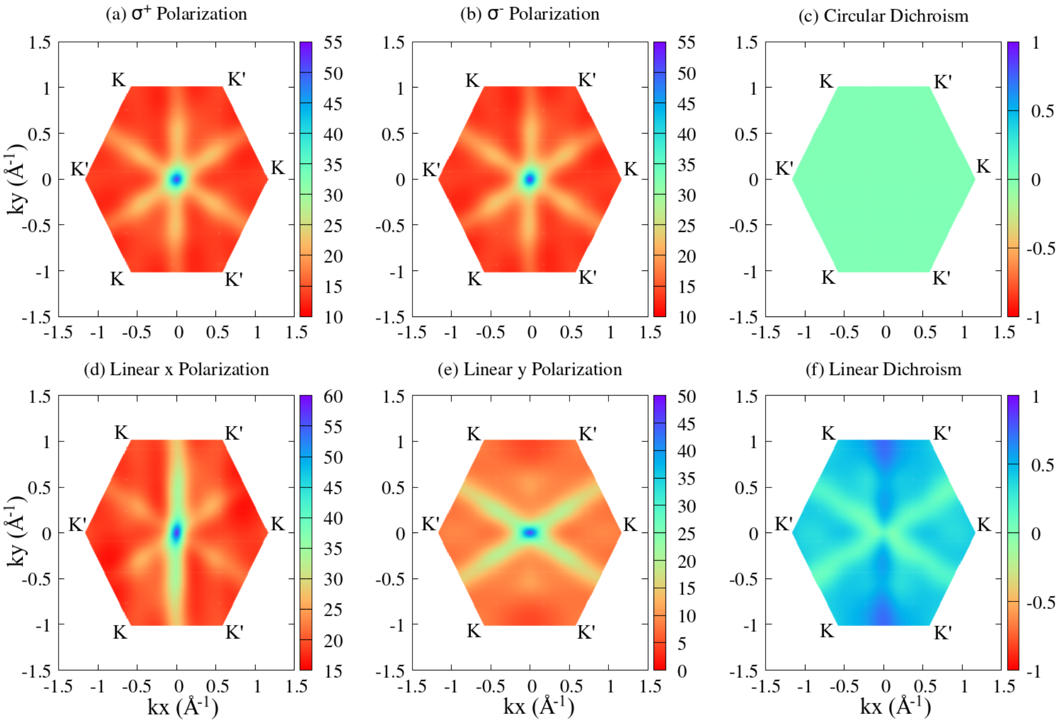

Figure S4: Optical activity of arsenene in the first BZ with circular light polarization in (a) and (b), linear light polarization in (d) and (e), and circular (c) and linear (f) optical dichroism. All calculations were conducted using DFT-HSE06+SOC and MLWF-TB parametrization.

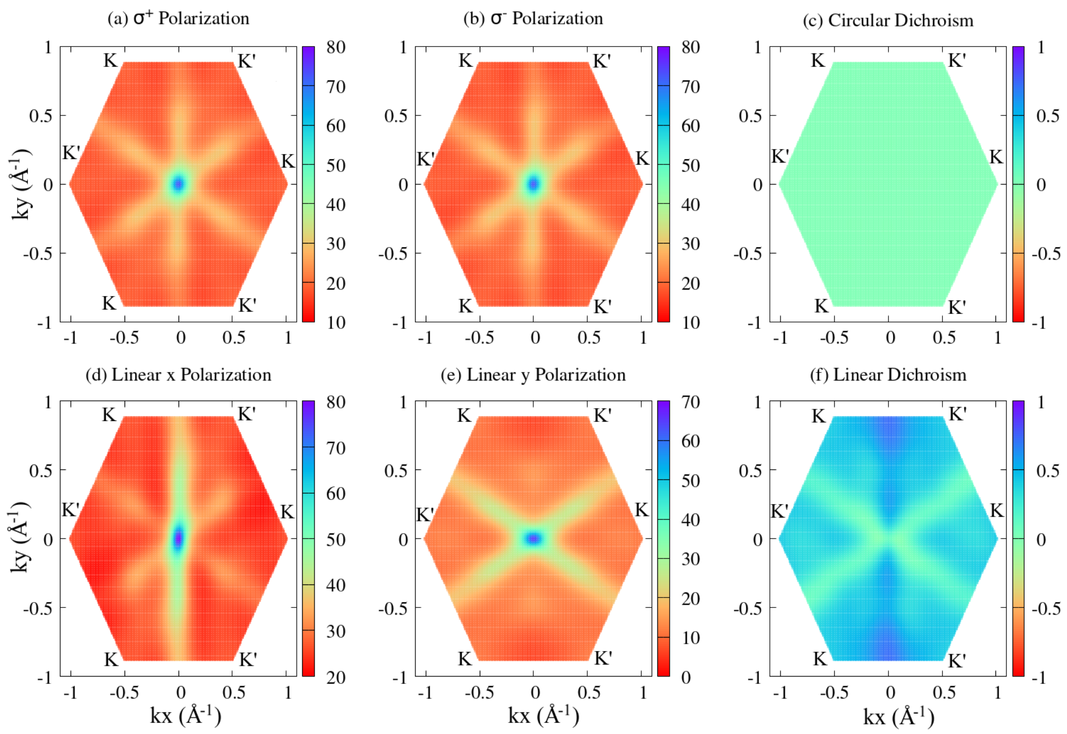

Figure S5: Optical activity of antimonene in the first BZ with circular light polarization in (a) and (b), linear light polarization in (d) and (e), and circular (c) and linear (f) optical dichroism. All calculations were conducted using DFT-HSE06+SOC and MLWF-TB parametrization.

5 BSE Simulation Parameters

Table S2: Parameters employed for Bethe-Salpeter Equation (BSE) simulations: the **k**-mesh, number of valence bands (n_v), number of conduction bands (n_c), and dielectric function smearing (η). All simulations utilized a Rytova Keldysh 2D potential (V2DRK),[?] with the system surrounded by vacuum (`LC=24,EDIEL_T=EDIEL_B=1.0`), as implemented in the WanTiBEXOS package,[?] where ε (`EDIEL`) represents the effective dielectric screening parameter.

System	**k**-mesh	n_v	n_c	η (eV)	ε
Nitrogenene	$50 \times 50 \times 1$	6	6	0.05	1.67
Phosphorene	$42 \times 42 \times 1$	6	6	0.05	3.04
Arsenene	$38 \times 38 \times 1$	6	6	0.05	3.52
Antimonene	$34 \times 34 \times 1$	6	6	0.05	4.13
